# Exploring the Potential of Ambient AI for Inpatient Documentation: A Qualitative Study with Junior Doctors

**DOI:** 10.1007/s10916-026-02437-7

**Published:** 2026-07-08

**Authors:** Aisling Bracken, Sean Whelehan, Anita Rose Babu, Khalid Merghani, Eoin Sheehan, Iain Feeley

**Affiliations:** 1 Royal College of Surgeons Ireland (RCSI), 123 St. Stephen’s Green, Dublin, Ireland; 2 Midlands Regional Hospital Tullamore, Arden Rd, Tullamore, Offaly, Ireland; 3 School of Medicine, University of Limerick, Castletroy, Limerick, Ireland

**Keywords:** Ambient AI, Clinical Documentation, Qualitative Study

## Abstract

Clinical documentation is essential for safe and effective patient care but places a substantial clerical burden on doctors, particularly those early in training. Ambient artificial intelligence (AI) systems, which passively capture clinical conversations and generate structured notes, have demonstrated promise in outpatient and primary care settings. However, their use in inpatient ward rounds remains largely unexplored. Semi-structured interviews were conducted with ten postgraduate year one doctors following participation in simulated orthopaedic ward rounds incorporating an ambient AI scribe (Heidi Health, Melbourne, Australia). The topic guide was informed by Normalisation Process Theory (NPT) to explore participants’ understanding of AI-assisted documentation workflows, the work they anticipated would be required at individual, team, and system levels for implementation, and their appraisal of the potential benefits, risks, and practical challenges of future use. Data were analysed collaboratively by two researchers using reflexive thematic analysis with a hybrid inductive–deductive approach. Seven themes were identified and mapped across the four NPT constructs. Coherence was reflected in participants’ understanding of ambient AI as a way to reduce documentation burden, support patient-centred records, and improve accessibility. Cognitive participation was evident in their recognition that implementation would require individual engagement, including review and approval of AI-generated outputs. Collective action captured the practical work required to adapt ward round behaviours, redistribute documentation tasks, manage consent, and address infrastructure, access, and governance requirements. Reflexive monitoring was demonstrated through participants’ appraisal of anticipated benefits, including potential for improved clinical engagement, multidisciplinary communication, and real-time capture of complex conversations, balanced against risks of clinical misrepresentation, cultural resistance, and privacy concerns. Through simulated exposure, participants made sense of ambient AI scribes as a potentially valuable addition to inpatient documentation workflows, anticipating effects on efficiency, communication, and patient-centred documentation. However, they also identified individual, team, and organisational work required for implementation, including workflow adaptation, consent processes, data privacy, access controls, infrastructure, and real-world appraisal in clinical settings.

**Supplementary Information:**

The online version contains supplementary material available at 10.1007/s10916-026-02437-7.

## Introduction

Clinical documentation is fundamental to safe and effective patient care, providing a key mode of communication between healthcare providers, a record for medicolegal purposes, and a means of ensuring coordinated care [1, 2]. Patient-oriented documentation consumes a substantial proportion of doctors’ working hours, often extending into out-of-hours periods, and has been shown to frequently exceed the time spent in direct patient care [3–5]. While the necessity of patient orientated documentation is undisputed, clerical responsibilities have been increasingly associated with job dissatisfaction and burnout [6] This burden is particularly acute for early career doctors, who undertake a disproportionate share of documentation and administrative tasks compared with senior colleagues [5].

Artificial intelligence (AI) powered systems represent the newest addition to the digital health repertoire. Recent advances in ambient AI have created new opportunities to reduce the clerical burden of documentation. Ambient documentation systems function as virtual scribes, positioned to operate unobtrusively in the background and passively capture clinical conversations to generate structured records in real time. Emerging evidence from outpatient and primary care contexts, suggests that these systems may improve documentation efficiency and the quality of clinical notes, while reducing the perceived workload associated with documentation tasks [7–9]. However, there is an absence of literature on the use of this technology in the inpatient setting.

Ward rounds are a focal point of inpatient care, providing opportunity to establish diagnoses, refine management plans and formulate discharge procedures, all of which must be documented [10, 11]. Orthopaedic ward rounds, in particular, are complex and fast-paced, reflecting the need to balance the care of large numbers of patients with the demands of operative scheduling [11]. In this high turnover environment, efficient and comprehensive documentation is critical, but remains a recognised challenge [11].

The introduction of an ambient AI scribe into the inpatient documentation workflow may alleviate clerical workload, however it would also represent a significant change to ward round delivery and established documentation practices. The successful implementation of such technologies hinges not only on technical performance but also on stakeholder input and acceptance [12]. The present study explored junior doctors’ perceptions of an ambient AI documentation system within simulated orthopaedic ward rounds, to examine how the technology was understood, engaged with, operationalised, and appraised as a potential component of inpatient documentation practice.

## Methods

### Study Design and Context

The study is reported in accordance with the Consolidated Criteria for Reporting Qualitative Research (COREQ) checklist. This qualitative analysis was conducted alongside a quantitative evaluation of the AI system [13]. To provide participants with a realistic and standardised basis for reflection, interviews were conducted following a series of simulated orthopaedic ward rounds. The simulations were designed to reproduce the pace and complexity of real clinical ward rounds. As part of the ward round simulation procedure each junior doctor was provided with a 10-minute demonstration on the use of the chosen ambient documentation system, Heidi Health (Melbourne, Australia.) prior to use in the simulation. Heidi Health Pro was utilised during the study. Templates for progress notes and discharge summaries were designed in accordance with local institutional documentation standards and were used to guide note generation during the simulated ward-round encounters.

### Participants and Recruitment

A purposive sampling strategy was employed. Eligible participants were postgraduate year one (PGY-1) junior doctors completing a three-month rotation in orthopaedic surgery at the study site. In our institution, inpatient documentation practices, including daily ward round notes and general practitioner discharge summaries, are completed exclusively by PGY-1 doctors. Ten junior doctors were recruited and interviewed. Seven participants had taken part in the initial simulated ward-round study used for the quantitative assessment of ambient AI documentation [13]. A further three junior doctors were recruited to participate in the simulation and interview process to add depth to the qualitative analysis. These additional participants did not contribute data to the quantitative assessment. All ten interviews were included in the final analysis. In line with reflexive thematic analysis principles of recruitment adequacy was considered using the concept of information power rather than data saturation. The study aim was relatively narrow and sample specificity was high, as participants were drawn from a single professional group and had all undergone the same simulated exposure prior to interview. Normalisation Process Theory (NPT) provided an established theoretical framework to inform both data collection and interpretation. Dialogue quality was assessed iteratively during data collection which demonstrated relevant reflections on participants’ experiences. Collectively, these factors indicated that the sample provided sufficient information power to address the study aim.

### Data Collection

Implementation of an ambient AI scribe in the inpatient setting represents a significant departure from routine documentation practices and requires not only technical accuracy but also behavioural, cultural, and organisational adaptation. The interview topic guide was informed by NPT. The four constructs of NPT; coherence, cognitive participation, collective action, and reflexive monitoring shaped development of the guide and aimed to sensitise analysis to the individual and organisational work anticipated to be required by junior doctors to integrate ambient AI documentation into inpatient practice. Individual semi-structured interviews were conducted by a single researcher (A.B, Female) who had no prior supervisory or evaluative relationship with participants. Each participant had taken part in multiple ward round simulations, acting at least once as clinical lead and once as controller of the ambient AI scribe. Participants were aware that the interviewer had a specific research interest in clinical documentation practices and evaluating the use of novel technologies to support inpatient workflows. Following completion of all simulations, interviews were undertaken in a private office setting to ensure impressions were captured at source. All interviews lasted between 20 and 30 mins and were conducted in person. Only the researcher (A.B) and participant were present during interviews. Interviews were audio-recorded with consent and transcribed verbatim, field notes were taken by the researcher during interviews to capture non-verbal findings. All participants were provided with an opportunity to review and amend their interview transcripts for accuracy prior to analysis. Participants did not provide feedback on the final themes. No repeat interviews were conducted.

### Data Analysis

Analysis was undertaken using Braun and Clarke’s reflexive thematic analysis [14]. A hybrid inductive–deductive approach was employed: inductive coding allowed novel concepts to emerge from the data, while NPT provided a sensitising framework to identify broadly held perceptions that would influence future implementation. The analytic process was collaborative, with two researchers involved in coding and theme development to support a broadened interpretation of the data. Coding was conducted manually, coding software was not utilised. Both researchers first read and reread all transcripts to become familiar with the dataset. Two transcripts were then independently coded line by line by both researchers. Coding was carried out at both semantic and latent levels, capturing explicit accounts as well as underlying assumptions and meanings. Following this initial coding, the researchers met to compare codes, discuss interpretations, and develop a provisional coding framework. Codes with overlapping or closely related meanings were reviewed and, where appropriate, refined into a single shared code.

A provisional coding framework was created to provide a flexible basis for the developing analysis, rather than to facilitate intercoder agreement. The coding framework was subsequently applied to the remaining transcripts. Coding remained iterative, with reviewers discussing new codes identified during analysis of each subsequent transcript and determining whether these should be added to the framework. All transcripts were coded by both reviewers. The final coding framework reflected the perspectives and interpretations of both reviewers. The final coding framework was used to identify overarching themes and subthemes by examining patterns across the dataset and relationships between codes. Themes were refined through discussion between the two researchers, with consideration of internal coherence, distinction between themes, and relevance to the research question.

Reflexivity was considered throughout the study. The interviewer (A.B.) is a medical doctor (BMBS, B.Sc.) undertaking a higher research degree (MD), with a primary research interest in AI-powered clinical documentation systems. As part of their higher degree training, they developed experience in qualitative research methods, including semi-structured interviewing and reflexive thematic analysis. Given the research team’s interest in the potential of ambient AI to reduce documentation burden, the possibility of favourable assumptions influencing data collection and interpretation was acknowledged.

## Results

Ten junior doctors participated in semi-structured interviews following a series of simulated ward rounds using an ambient AI scribe. All participants were postgraduate year one doctors undertaking a three-month orthopaedic rotation. The sample included six male and four female participants, with an age range of 23–31 years. One participant reported prior awareness of ambient AI scribes, although none had previously used the technology. All participants reported prior use of AI tools, specifically large language models, outside of the workplace.

Using NPT as an analytical framework, seven overarching themes were identified. These themes reflected how participants understood the role of an ambient AI-assisted workflow in the context of their current administrative burden (coherence), the individual, team, and organisational work they anticipated would be required for future integration (cognitive participation and collective action), and their appraisal of the potential implications of real-world use (reflexive monitoring). 

### 1. Timesaving

This theme broadly reflects the NPT construct of coherence. Participants viewed an ambient scribe as a potential response to the recognised burden of inpatient documentation, despite most having no prior awareness or experience of ambient scribes before the study. Within the simulated ward round context, an ambient scribe was perceived as a way to reduce retrospective burden and streamline workflow allowing time saved to be redirected towards patient-facing clinical tasks. Cognitive participation was reflected in the subtheme of task redistribution, as participants considered the work that would be required on a personal level to engage with and integrate the technology successfully (Table 1).

**Table 1:** Participant Quotes on the Theme of Timesaving

Time – Saving	Elimination of Retrospective Burden	‘We discharge quite a few patients every day, it’s almost impossible to stay on top of the discharge summaries. There’s always a backlog’ - *Participant 4* ‘Complex discharge summaries can take 30 mins plus, when you have to discharge 3 or 4 patients a day, sometimes more, on top of writing ward round notes it really adds up. This [ambient AI] would be a massive help.’ - *Participant 6*
	Uninterrupted & Streamlined Workflow	‘Writing updates from the round can take hours, it can be the afternoon before I finish because there are always interruptions or other jobs to do’ - *Participant 1*
	Redistribution of documentation tasks	‘Using this [ambient AI] creates another job someone has to check the notes to make sure they’re right… I still think it will be quicker than having to write all the notes the way we do now’ - *Participant 2* ‘One intern could check and print the [AI generated] notes – this would free up the other interns to get urgent jobs done, like following up patients who were unwell overnight without worrying about writing 15 notes from the round ’ - *Participant 6* *“*We still use paper charts, so it’s not a matter of just direct copy and paste into an EHR. Someone will have to print the notes so they can be filed’ - *Participant 5*

#### Elimination of Retrospective Burden

Current documentation workflows were described as “time-consuming,” “retros

pective,” “tiring” “frustrating” and creating “backlogs”. In contrast, participants saw ambient AI as a fundamentally new way of working, offering immediate progress note availability and a mechanism to produce more timely discharge summary documentation.

### Uninterrupted and Streamlined Workflow

Junior doctors described frequent delays in documentation caused by competing clinical demands and logistical challenges such as locating patient charts. Progress notes were often reported as being completed hours later, which participants viewed as diminishing their usefulness for daily planning and timely communication with the wider team. In contrast, the ambient scribe was viewed as a means to potentially restore immediacy to documentation, transforming progress notes from retrospective records into real-time clinical tools that could directly support patient care decisions.

#### Redistribution of Documentation Tasks

Participants acknowledged that while an ambient AI documentation system would introduce the new responsibility of reviewing and validating generated notes, this was viewed by some participants as a worthwhile trade-off. Participants suggested the technology may have the potential to reduce duplication of effort among junior doctors. Participants envisaged a system where instead of every doctor documenting separately, one individual would take responsibility for checking and printing the AI-generated notes, allowing other doctors to focus on urgent clinical tasks. This redistribution was perceived as a way to save time collectively and improve the efficiency of ward round follow-up. Junior doctors additionally identified the continued reliance on paper-based records as a potential obstacle to smooth integration, as AI-generated notes would still need to be printed and filed, creating additional steps in the workflow. This practical limitation was recognised as unique to technologically less mature environments and was viewed by participants to undermine the perceived efficiency gains associated with the system.

### 2. Accuracy and Reliability

This theme aligns with the NPT construct of reflexive monitoring. Drawing on their exposure to AI-generated ward round notes during the simulation, participants appraised the potential consequences of using AI-generated documentation in clinical practice. Their evaluation was balanced: perceived benefits in detail, legibility, and capture of relevant clinical information were weighed against concerns regarding clinical misrepresentation. The subtheme of human oversight and supervision also reflects cognitive participation, as junior doctors anticipated an individual responsibility to review, edit, and approve all AI-generated outputs (Table 2).

**Table 2:** Participant Quotes on the Theme of Accuracy and Reliability

Accuracy & Reliability	Human Oversight and Supervision	“Overall, it is [ambient AI] quite impressive, it provides a level of detail that I think would be hard to capture even if I was trying to write the note while at the patient’s bedside.” *- Participant 4* “It’s not at the level that you could trust it solely. You need to go back and review everything before it can be used.” *- Participant 1*
	Selective Capture of Relevant Information	“There was a point when the bleep was going off and the background noise was quite loud that I couldn’t hear myself think, I was really surprised that there was nothing extra from that noise in the note.” - *Participant 2*
	Risk of Clinical Misrepresentation	“Misspelling medications, documenting the wrong dose or giving non-specific follow up time could lead to problems. All of these would have to be checked before the note is approved.” - *Participant 1* “In the notes I reviewed there were no major errors, just some spelling mistakes. For example it wrote axial dressing instead of aquacel dressing.” *- Participant 4* “Even without editing I think having a couple of typos is better than having an illegible note that no one can read.” - *Participant 7*

#### Human Oversight and Supervision

Participants emphasised that ambient AI documentation could not be relied upon in isolation. While the system was viewed as detailed and impressive, junior doctors recognised that it was not infallible, having witnessed errors in documents generated during the simulation. This reinforced the perceived need for human review to ensure clinical safety and accuracy. The technology was therefore understood as an adjunct to clinician-led documentation, rather than a replacement for clinical judgement or an independently functioning system.

#### Selective Capture of Relevant Information

A positive feature of the system noted by participants was its ability to filter extraneous input and capture only clinically relevant discussion. Participants were surprised by the accuracy of transcription even in noisy environments. This ability to manage background interruptions was viewed as supporting the reliability of generated documentation.

#### Risk of Clinical Misrepresentation

Despite overall confidence in the system, junior doctors identified specific risks related to clinical misrepresentation. Errors such as medication misspellings, incorrect doses, or vague follow-up instructions were all highlighted as potential safety concerns, reinforcing the requirement for human oversight and supervision. Participants contrasted these risks with the limitations of current handwritten documentation. Even when AI-generated notes contained typographical errors, they were considered by some participants as more legible and clinically useful than some existing records.

### 3. Clinical Communication and Collaboration

This theme aligns primarily with the NPT construct of reflexive monitoring. Participants anticipated that ambient scribes could potentially improve communication with the wider multidisciplinary team by making clinical updates and management plans available in a more timely and accessible manner. This appraisal reflected participants’ evaluation of how AI-generated documentation might influence team-based workflow, continuity of care, and the sharing of clinical information beyond the immediate ward round team (Table 3).

**Table 3:** Participant Quotes on the Theme of Clinical Communication & Collaboration

Clinical Communication & Collaboration	Facilitating Multidisciplinary Communication	“I often end up giving the nurses or physios verbal updates on patient plans because I haven’t had the chance to write the ward round note, …, if using the AI meant the notes were done, everyone would know the plan from 8am.” -*Participant 4*
	Coordination in Clinical Decision-Making	“The ward round is divided between the orthopaedic ward and outliers, even if someone wasn’t on one part of the round, they could log in and see exactly what was said and decided.” - *Participant 8*

#### Facilitating Multidisciplinary Communication

Participants emphasised that real-time documentation held potential to strengthen communication across the multidisciplinary team. In current practice, delays in writing notes often left nurses, physiotherapists, and other allied health professionals reliant on fragmented or delayed verbal updates, creating risks for continuity of care. By generating documentation in real time, ambient AI was seen as providing immediate clarity on management plans and decisions, ensuring that all team members were aligned from the start of the day. In line with the identified subtheme of streamlined workflow, participants felt that this reduced the need for repeated explanations and freed doctors to redirect their focus towards patient care and urgent clinical tasks.

#### Coordination in Clinical Decision-Making

AI-generated notes were viewed as enhancing transparency across the team, particularly when ward rounds were divided across multiple locations.

### 4. Usability and Accessibility

This theme aligns primarily with collective action, as participants considered the practical work required at team and organisational levels to implement ambient AI scribes in ward based practice. Drawing on simulated use, participants viewed the system as intuitive, portable, and easy to operate, with mobile access lowering barriers to adoption. However, they identified potential real-world implementation factors not assessed in simulation, including internet connectivity and infection control requirements, which may hinder adoption. Coherence was also reflected in one participant’s experience of the scribe as an accessibility aid, broadening its perceived value beyond efficiency alone (Table 4).

**Table 4:** Participant Quotes on the Theme of Usability & Accessibility

Usability and Accessibility	User-Friendly Interface and Portability	“It was easy to use, it’s very handy that we can access it through an app on our phones.” – *Participant 10*
	Ward Infrastructure	*“For patients in isolation rooms it might not be as straightforward – someone would probably have to stand at the door and it’s hard to know will it work as well in this scenario” - Participant 3* ‘We’re going to be dependent on the Wi-Fi working properly on the ward. If the internet is slow that’ll slow the round down’ *- Participant 5*
	Accessibility for Diverse Users	*“As someone with a hearing impairment … this acts as another set of ears*,* even with distractions and background noise it seemed to capture pretty much everything that was said”* - *Participant 3*

#### User-Friendly Interface and Portability

The system’s ease of use was identified as a benefit by participants, who described it as intuitive, straightforward, and easy to use. Junior doctors reported a modest learning curve with the technology. The availability of a mobile application was considered particularly valuable, as it allowed doctors to access and activate the system without reliance on fixed workstations. This portability was seen as aligning well with the realities of ward-based work. The simplicity of “one-click” functionality was interpreted as lowering the barrier to adoption, as it required little additional training or cognitive effort.

#### Ward Infrastructure

Despite acknowledgement of the system’s ease of use, participants identified several environmental and logistical factors that might limit its effectiveness in ward round practice. Use in isolation rooms, particularly for patients with multi-drug resistant organisms, was described as a potential challenge, as equipment could not always be brought into patient areas. Participants suggested workarounds, such as positioning devices at the doorway, but recognised that this could compromise accuracy. Reliance on adequate internet connectivity while moving throughout wards was also highlighted as a potential barrier, with concerns that connectivity issues might interrupt or slow the pace of ward rounds.

#### Accessibility for Diverse Users

The Ambient AI scribe was identified to offer accessibility benefits that could support a wider range of users. One participant with hearing impairment, who described the system as providing reassurance by acting as an “extra set of ears” during busy ward rounds. This inclusivity was interpreted as extending the potential value of ambient AI beyond efficiency alone, making clinical documentation more equitable, benefiting both clinician and patient.

### 5. Adapting Ward Round Practice

This theme aligns primarily with collective action, as participants drew on their simulated experience to consider the team and organisational level work required to integrate ambient scribes into real-world practice. They recognised that effective use would require adaptation of existing team behaviours. They anticipated that inconsistent patient consent and cultural resistance, could potentially disrupt workflow and hinder integration into routine practice (Table 5).

**Table 5:** Participant Quotes on the Theme of Adapting Ward Round Practice

Adapting Ward Round Practice	Behavioural Adaptation	*“Announcing the early warning score or if the patients are on antibiotics will take some getting used to… I think this change would probably improve the quality of the round though.”* - *Participant 2* ‘The way we do the round at the moment not everything is said out loud, so this would have to change for it to work effectively, it wouldn’t be a major thing to change but I think it would need to be a conscious effort’ - *Participant 1* ‘I don’t know if everyone would be happy to have a device listening in the background, like it might be more awkward to use it if only some people are consenting to it’ - *Participant 7*
	Cultural Resistance	“I think everyone is so used to the way things are done, for this to work people will have to be open to changing the way we run the round” *- Participant 1*

#### Behavioural Adaptation

Participants acknowledged that ambient AI would require changes in how ward rounds were conducted, particularly in relation to verbal communication. At present, not every clinical decision or observation is explicitly verbalised during rounds, as much is understood implicitly within teams. For the system to work effectively, participants recognised that more structured and deliberate verbalisation of information would be necessary. While this was seen as an adjustment, many felt it could also improve clarity and inclusiveness in clinical decision-making. Concerns were also raised that inconsistent patient consent could fragment ward rounds, complicating workflow.

#### Cultural Resistance

Several participants anticipated cultural resistance to the introduction of ambient AI, particularly among senior colleagues who might perceive less direct benefit from its use. This hierarchical divide was highlighted as a potential barrier to embedding the technology within routine practice.

### 6. Patient-Centred Documentation

This theme aligns primarily with coherence, as participants understood ambient AI scribes as a means of improving the quality and patient-centredness of clinical documentation. Drawing on their simulated experience, participants described real-time capture as a mechanism to preserve the detail of complex conversations. The subtheme of detailed capture of patient interactions also reflects reflexive monitoring, as participants appraised how AI-generated documentation could enhance the fidelity of the medical record (Table 6).

**Table 6:** Participant Quotes on the Theme of Patient Centred Documentation

Patient-Centred Documentation	Detailed Capture of Patient Interactions	‘If the reg explains a procedure and the specific risks to a patient, it’s very hard to accurately document this retrospectively, with this doing the note, all those details will be included’ – *Participant 10* ‘it can be hard to keep track of every aspect of the interaction so while I might write down their EWS, I might miss their mobility status, this documents everything’ - *Participant 3*
	Deeper Clinical Engagement	‘I have also worked jobs where I wrote the note at the bedside, it’s very difficult to focus on the patient and write everything down at the same time. Using this in the background would mean that you could just focus on the patient and I think the detail in the note would be better’ – *Participant 9* If we had freer time and we weren’t doing this [manual documentation], I think we would have more time to interact with patients, interact with family members’ - *Participant 6*

#### Detailed Capture of Patient Interactions

Participants emphasised that a valuable aspect of ambient AI was its ability to capture the richness of patient clinician interactions in ways that traditional note-taking often missed. They highlighted that during busy ward rounds, conversations about treatment plans, surgical risks, and patient concerns were rarely documented in full, particularly when notes were written retrospectively. The system’s capacity to record these details in real time was seen as enhancing the fidelity of the medical record.

#### Deeper Clinical Engagement

Participants felt that ambient AI could free them from the cognitive and physical task of note-taking at the bedside, enabling more meaningful patient interactions. Writing notes in real time was described as distracting, often pulling attention away from patients and undermining the quality of the clinical encounter. By allowing doctors to focus on communication and patient assessment, ambient AI was perceived as supporting deeper engagement. Participants framed these benefits as directly tied to the time saved by reducing clerical workload, potentially providing them with more time for direct patient care.

### 7. Privacy and Confidentiality

This theme aligns primarily with collective action, as participants considered the ethical, practical, and governance work required to implement ambient AI scribes safely in inpatient care. Patient consent, decision-making capacity, access controls, and data storage were identified as key factors that would need to be addressed before routine use (Table 7).

**Table 7:** Participant Quotes on the Theme of Privacy & Confidentiality

Privacy & Confidentiality	Patient Consent	‘There are some patients who are admitted to the ward who probably don’t have capacity to consent’ - *Participant 5*
	Access Controls and Data Governance	“It’s a question of who has access to the app, this would have to be monitored.” - *Participant 5* “I think some people get wary when they hear artificial intelligence, like I would want to be sure that my personal data isn’t being used to train the AI model.” - *Participant 7*

#### Patient Consent

Concerns were raised about how consent for ambient AI use would be obtained and managed in the inpatient setting. A participant highlighted particular challenges with patients who lacked decision-making capacity, such as those with dementia or acute delirium, where explicit consent might not be feasible. This was seen as raising ethical and practical questions about whether the technology could be applied uniformly across the ward or whether alternative strategies would be required. Participants also reflected that applying the system selectively could create inconsistencies in documentation practices, potentially undermining some of its perceived benefits.

#### Access Controls and Data Governance

Participants emphasised the need for strict oversight regarding who could access AI-generated notes and how data would be stored or shared. Concerns about information governance were closely linked to broader questions of patient trust. The terminology of “artificial intelligence” itself was seen as potentially problematic, with some patients and staff likely to be sceptical or anxious about how their data might be used. These concerns highlighted that alongside technical and workflow integration, successful adoption would depend on transparent communication about privacy protections and robust governance frameworks.

## Discussion

Successful and sustained integration of new technology into clinical practice depends on more than technical performance; it requires users to understand its purpose, adapt everyday work around it, judge its value in practice, and be supported by organisational structures that enable its routine use. These processes are represented in the constructs of NPT [15]. Junior doctors understood ambient AI scribes as a plausible response to the clerical burden of inpatient documentation, particularly where documentation workload affected efficiency, communication, and patient-centred care. Their willingness to engage with the technology was tempered by recognition that safe use would require active review, approval, and incorporation of AI-generated outputs into existing clinical responsibilities, with potential to introduce additional workload. Participants also recognised that implementation would require wider changes to ward round practice, team communication, infrastructure, consent processes, and governance. Overall, these findings suggest that while junior doctors perceived ambient AI scribes as potentially valuable, routine use in inpatient care would depend on whether the technology can be embedded within existing clinical cultures, supported by senior clinicians, and governed through clear organisational processes.

The time-saving benefits perceived by junior doctors in this study mirror findings from both primary and ambulatory care. In early NHS implementations of ambient systems including Heidi Health, the platform trialled in this study, general practitioners have similarly reported improvements in efficiency, reduced cognitive demand, and enhanced documentation quality [16]. Crucially, across these settings, a consistent theme has been the opportunity to redirect attention from clerical work back to the patient [7, 16]. Similarly, junior doctors in this study anticipated that ambient AI would enable more meaningful patient interactions by freeing time otherwise spent on retrospective documentation.

Following exposure to ambient scribe use in the simulated environment participants also identified the potential of the scribe to expedite discharge summary documentation. In this study, junior doctors described discharge summaries as particularly burdensome, with volume and patient turnover contributing to frequent delays in completion. Discharge summaries are a critical communication tool between hospital and community care, yet competing clinical demands often lead to their deprioritisation, resulting in delays and variable quality [17]. Given their central role in supporting continuity of care, improvement in discharge summary timeliness may have important clinical benefits [18]. Ambient AI was perceived as a way of streamlining this process. These perspectives align with emerging evidence that AI-assisted approaches can improve the quality and timeliness of discharge communication, with potential implications for both patient safety and system efficiency [17, 19].

During the linked quantitative component of the study, junior doctors reviewed and approved AI-generated ward round documentation, providing them with direct experience on which to assess the accuracy and content of the outputs [13]. Participants emphasised that while the system was capable of producing detailed and timely documentation, clinical information, particularly medications, dosages, and follow-up plans, was not always accurate and required human review before being used in patient care. Wider literature on AI in healthcare emphasises that AI-generated documentation is not infallible [20]. The medico-legal implications of errors in AI-assisted documentation remain uncertain, particularly where documentation has been generated by an AI system but incorporated into the clinical record by a clinician. The NHS has released guidance which highlights the importance of transparent review processes, traceable clinician approval, and clearly defined accountability for AI-generated documentation [21]. Participants suggested that oversight tasks, such as reviewing and signing off notes, could be integrated into existing workflows without negating efficiency gains, especially if distributed across the team. Framing the technology as an adjunct rather than a replacement for doctor responsibility was seen as essential for its acceptability.

Junior doctors anticipated that integrating ambient AI into ward round practice would require behavioural adaptation, which they perceived as a potential barrier to adoption. This was understood not only as a technological shift, but also as a predicted change to established clinical routines. One anticipated adjustment involved more deliberate verbalisation of information that is not traditionally spoken aloud during ward rounds. Although this was expected to require adjustment, participants suggested that it could potentially improve the quality of the ward round. This perceived opportunity to strengthen ward round practice emerged as an unanticipated potential benefit. Reframing the ward round as a space where clinical information is more explicitly articulated was expected to promote clarity in management for both patients and the wider team. The potential to strengthen ward rounds in this way is notable, as poor-quality rounds have been linked to patient complaints, delayed discharges, and increased hospital costs [10, 22, 23]. Despite this, ward rounds have historically been overlooked as targets for structured improvement [10].

Beyond behavioural adaptation and the work required at an individual level, participants identified several organisational requirements for real-world implementation of ambient AI scribes in inpatient care. These included governance around the use and privacy of recorded data, adequacy of supporting technological infrastructure, and clear processes for obtaining and managing patient consent. These concerns mirror those highlighted in recent literature [24]. Clear communication about what data is collected, how it is stored, and the measures taken to mitigate bias is essential for maintaining trust [25]. From a legal standpoint, implementation must comply with frameworks such as the GDPR [25]. In the inpatient setting, an additional consideration is that a proportion of patients may lack capacity to provide consent, raising the question about timing of consent, and how best to manage situations where capacity fluctuates. Participants noted in light of this that some encounters may not be appropriate for ambient recording, necessitating clear protocols and alternative documentation pathways for patients who opt out. Technical challenges, including consistent connectivity while mobile on a ward round, were also recognised as critical to effective adoption. Addressing the ethical, legal, and technical considerations through governance frameworks, collaborative policy development, and investment in infrastructure will be key to enabling safe, compliant, and sustainable integration of an ambient AI scribe into real world inpatient care.

The study also highlights broader sociocultural considerations relevant to the normalisation of ambient AI in clinical practice. Junior doctors appeared to make sense of the technology not only as a documentation aid, but as a potential support for team-based communication, accessibility, and participation during ward rounds. However, they acknowledged that this understanding may not be shared equally across professional groups, particularly among senior colleagues or members of the multidisciplinary team who may perceive fewer direct advantages. As ward rounds are inherently team-based, successful integration will require shared ownership across professional groups, not just among junior staff. The experience of one participant with a hearing impairment further broadened how the technology was understood, suggesting that an ambient scribe may have value beyond efficiency by supporting more inclusive participation in ward-based clinical work.

This study has several limitations. The simulated environment was deliberately chosen as an exploratory mechanism to allow junior doctors to engage with ambient AI scribes in a controlled, low-risk setting before real-world clinical implementation. While this enabled participants to reflect on its use without affecting patient care or routine ward workflow, simulation may not fully capture the complexity of live ward rounds. The study was conducted with a relatively small number of junior doctors. Recruitment adequacy was considered in relation to information power rather than data saturation; however, the sample size remains a limitation, and findings should be interpreted as exploratory. The exclusive focus on junior doctors, while relevant given their central role in inpatient documentation, means that the perspectives of the broader clinical team were not captured.

## Conclusion

Following simulated exposure, junior doctors understood ambient AI scribes as a potentially credible addition to inpatient documentation workflows. Participants anticipated that implementation would require active engagement at an individual level, including review, approval, and incorporation of AI-generated outputs into existing clinical responsibilities. They also identified wider team and organisational work required to embed the technology into ward round practice, including adaptation of clinical routines, infrastructure, governance, consent processes, and senior clinician engagement. Junior doctors’ appraisal of ambient AI was therefore shaped not only by its technical function, but by the practical, cultural, and organisational conditions required for its normalisation in real-world inpatient care.

## Supplementary Information

Below is the link to the electronic supplementary material.

Supplementary Material 1

## Data Availability

Research data is available upon reasonable request.
